# Fluorine-Free and Waterborne: A Sustainable Polyurethane Coating Strategy for Superhydrophobic and Anti-Graffiti Fabrics

**DOI:** 10.3390/polym18172063

**Published:** 2026-08-25

**Authors:** Jing Yang, Shengzhe Hou, Ziyi Li, Yan Zhao

**Affiliations:** 1 College of Textile and Clothing Engineering, National Engineering Laboratory for Modern Silk, Soochow University, Suzhou 215123, China; 2 Jiangsu Advanced Textile Engineering Technology Center, Nantong 226007, China

**Keywords:** superhydrophobic fabric, polyurethane coating, anti-graffiti, liquid-like surface, polydimethylsiloxane

## Abstract

Liquid-like surfaces are constructed by anchoring flexible polymer chains with low glass-transition temperature onto solid substrates, imparting low liquid adhesion and easy sliding. Despite their considerable promise in anti-fouling and anti-adhesion applications, such surfaces have thus far been mainly demonstrated on smooth planar substrates. In this work, we report a waterborne polyurethane superhydrophobic fabric coating that features a liquid-like surface behavior and anti-graffiti functionality. The coating was prepared by incorporating dihydroxy single-end-terminated polydimethylsiloxane (PDMS) into polyurethane backbone, which not only reduces the surface energy of the resulting coating but also allows the formation of PDMS brushes at the coating interface, owing to the orientation and enrichment of PDMS moieties at the surface upon coating formation. The as-prepared coating on cotton and polyester fabrics exhibited static WCAs of 158.4° and 157.3°, with WSAs of 9.1° and 7.9°, respectively. Moreover, the coated fabrics demonstrate excellent anti-graffiti performance and self-cleaning ability. Mechanical durability tests revealed that the coating retained a static WCA of approximately 150° even after 20 laundering cycles or 50 abrasion cycles. Given its waterborne and fluorine-free nature, this strategy offers a feasible pathway to extend liquid-like surfaces from smooth substrates to textile substrates for practical anti-graffiti and self-cleaning applications.

## 1. Introduction

Superhydrophobic fabrics have attracted extensive attention in the fields of healthcare, self-cleaning, outdoor apparel, and protective clothing [1,2,3]. Their distinctive features including water repellency, reduced fouling, and self-cleaning capability make them highly desirable for both daily use and specialized applications. The preparation of superhydrophobic fabrics generally relies on the modification of fiber surfaces with low-surface-energy materials. Fluorine-containing compounds, characterized by their exceptionally low surface energy, can simultaneously impart both superhydrophobicity and anti-fouling properties to textiles. However, such substances are resistant to degradation and exhibit bioaccumulation potential, posing toxicity risks to living organisms [4,5]. Consequently, their application in the textile industry has encountered increasing regulatory restrictions and even outright bans in many regions [6]. In contrast, fluorine-free low-surface-energy materials offer an environmentally friendly alternative. Typically, polydimethylsiloxane (PDMS), with a surface tension as low as 20 mN/m [7], has been widely used for fabricating superhydrophobic fabrics through simple dip-coating using a PDMS precursor solution [8,9,10,11], or via plasma-assisted coating processes [12,13,14]. Nevertheless, most of these PDMS-based coatings consist of cross-linked networks, and they typically confer only superhydrophobicity to the fabric surface, without providing effective anti-graffiti functionality.

In recent years, liquid-like surfaces have emerged as a promising platform in the fields of anti-fouling and anti-adhesion [15,16,17]. Such surfaces are typically fabricated by grafting flexible polymer chains that have ultralow glass-transition temperatures (*T*_g_), such as perfluoropolyether (PFPE) and PDMS with a *T*_g_ of −116 °C and −127 °C [18,19], respectively. At room temperature, the tethered polymer chains possess extremely low energy barriers for conformational transitions resulting from their low *T*_g_, thereby enabling the tethered chains to behave like an ultrathin liquid film [20,21,22]. This liquid-like dynamic behavior endows the surface with low contact angle hysteresis for both water and organic liquids, allowing them to readily slide off. As a result, liquid-like surfaces exhibit dynamically omniphobic property and excellent anti-adhesion performance. Specifically, PDMS brush-based liquid-like surfaces have been widely employed for anti-icing [23,24], anti-biofouling [25], and liquid anti-adhesion [26].

Usually, PDMS brushes can be prepared via either grafting-from [22,27,28,29] (surface-initiated polymerization of reactive silane monomers) or grafting-to [30] (covalent attachment of reactive PDMS polymer chains) approaches. The grafting-to method suffers from intrinsically low grafting density due to steric hindrance and excluded-volume effects from the pre-attached chains. The grafting-from approach, while affording higher grafting densities, often involves complex and time-consuming procedures. Moreover, both approaches require reactive groups on substrates and are not readily applicable to diverse, low-activity surfaces. Alternatively, bulk coatings with PDMS brush-like surface can be facilely prepared by constructing crosslinked networks or polymeric backbones bearing highly mobile PDMS side chains. For instance, Hu et al. [31] prepared PDMS-modified epoxy coatings by incorporating PDMS-grafted branched poly(ethylene imine) into a commercial epoxy formulation consisting of bisphenol A diglycidyl ether (DGEBA) and a hardener mixture (Jeffamine, triethanol amine, and piperazine). Besides epoxy, they also incorporated acyl chloride-terminated PDMS into polyurethane matrix [32], using a styrene/methacrylate oliogomer as the polyol component and a hexamethylene diisocyanate trimer (HDIT) as the polyisocyanate component. Tian et al. [33] synthesized a diol with amide groups and flexible PDMS through the click reaction of thiolactone with diamine and mono-ethenyl-terminated PDMS, and then prepared polyurethane with PDMS side chains by the reaction of isocyanate and diol. Similarly, hyperbranched polyurethane coatings were prepared by using HDIT, mono-hydroxyl-terminated PDMS, and castor oil-based hyperbranched polyol [34] or hydroxyl-terminated hyperbranched polyester [35]. These coatings bearing PDMS side chains exhibit dynamic liquid repellency (i.e., low sliding angles for water and oils) and effective anti-graffiti properties on planar substrates. Nevertheless, the preparation of these coatings involves the use of organic solvents, which undermines their environmental friendliness and limits large-scale application. Although there appeared several studies [36,37,38] that tried to prepare waterborne polyurethane coatings with PDMS side chains, these coatings have been confined to rigid planar substrates for super-slippery applications. The finishing requirements for superhydrophobic textiles, however, are quite different from those for rigid substrates, as superhydrophobic textiles necessitate not only a high degree of hydrophobicity but also superior softness and mechanical durability. Therefore, it is still highly desirable to develop waterborne coatings that are adaptable to the demanding requirements of textile finishing and capable of imparting both superhydrophobicity and anti-graffiti performance to fabrics.

Herein, we report the fabrication of a waterborne polyurethane coating that features a liquid-like surface behavior for superhydrophobic and anti-graffiti fabric coating applications. Polycaprolactone diol (PCL) was chosen as the soft segment for its hydrophobicity, which facilitates the achievement of superhydrophobicity in the final coating, and isophorone diisocyanate (IPDI) was employed as the hard segment building block. Meanwhile, dihydroxy single-end-terminated polydimethylsiloxane (S-PDMS) was covalently grafted into the polymer backbone via the reaction of its terminal hydroxyl groups with the isocyanate groups of IPDI, thereby resulting in the low-surface-energy PDMS side chains that are capable of migrating to the coating-air interface and forming the liquid-like surface. The influence of PDMS content on hydrophobic performance was investigated. The obtained coating was applied to both cotton and polyester fabrics, and the superhydrophobicity, self-cleaning ability, anti-graffiti performance, and mechanical durability of coated fabrics were evaluated.

## 2. Experimental Section

### 2.1. Materials

Dihydroxy single-end-terminated polydimethylsiloxane (S-PDMS) and hydroxy double-end-terminated polydimethylsiloxane (D-PDMS) were purchased from Anhui Aiyota Silicone Oil Co., Ltd. (Bengbu, China) and Guangzhou Slok Polymer Co., Ltd. (Guangzhou, China), respectively. Polycaprolactone diol (PCL) and dibutyltin dilaurate (DBTDL) were supplied by Shanghai Macklin Biochemical Technology Co., Ltd. (Shanghai, China). 2, 2-Bis(hydroxymethyl)propionic acid (DMPA) was obtained from Saen Chemical Technology Co., Ltd. (Shanghai, China). Triethylamine (TEA) was purchased from Beijing Innochem Technology Co., Ltd. (Beijing, China). Isophorone diisocyanate (IPDI) was acquired from Shanghai Bailingwei Chemical Technology Co., Ltd. (Shanghai, China). Cotton fabric (plain-woven, 206 g/m^2^, thickness: 0.278 mm) and polyester fabric (plain-woven, 210 g/m^2^, thickness: 0.319 mm) were purchased from a local store.

### 2.2. Preparation of the LSSiU Emulsions

First, a certain amount of S-PDMS was placed into a four-necked flask equipped with an overhead stirrer. Under nitrogen atmosphere, an appropriate amount of acetone was added, and the mixture was stirred for 10 min to ensure uniform mixing, then heated to 85 °C. The temperature was maintained at 85 °C, and 30 μL of DBTDL was added dropwise as a catalyst, followed by the dropwise addition of IPDI to initiate the reaction. After 30 min, PCL was added, and the reaction continued at 85 °C for another 30 min. Finally, DMPA was added and the reaction was allowed to proceed for 2 h. After the reaction was complete, the mixture was cooled to room temperature, and the neutralizing agent TEA was added for neutralization. Then, deionized water was added under high-speed dispersion, ultimately forming an LSSiU emulsion. The recipes used for preparing different LSSiU emulsions are listed in Table 1.

In addition, to compare the difference between single-end-anchoring and double-end-anchoring of PDMS chains on the anti-graffiti performance, in the LSSiU6 formulation, D-PDMS was used instead of S-PDMS to prepare a waterborne polyurethane emulsion as a control sample, and it was designated as LDSiU.

### 2.3. Synthesis of Silica Nanoparticles (SiO_2_ NPs)

To prepare the SiO_2_ NPs, 3 mL of water and 9 mL of ammonia solution were added to 150 mL of ethanol. Then, 4.5 mL of TEOS was added dropwise, and the solution was stirred at 40 °C for 4 h. Subsequently, another 3 mL of TEOS was added dropwise, and the temperature was maintained at 40 °C with continued stirring for 12 h. After the reaction, the solution was centrifuged, and the resulting precipitate was washed once with ethanol and twice with water. The obtained SiO_2_ NPs were stored for later use.

### 2.4. Preparation of the Coated Fabrics

A certain amount of the LSSiU emulsion was taken, and SiO_2_ NPs were added with stirring for 1 h. The solution was then ultrasonically dispersed for 30 min to obtain the desired finishing agent. Cotton and polyester fabrics were immersed in the above finishing agent for 1 min, then removed and placed in an oven at 120 °C for 30 min for drying and curing. After curing, the fabrics were taken out, washed with acetone, and dried at 60 °C to obtain the coated fabrics.

### 2.5. Characterizations

The particle size distribution of the waterborne polyurethane emulsion was measured by dynamic light scattering (DLS) using a Malvern Zetasizer Nano ZS90 analyzer (Malvern Instruments Ltd., Malvern, UK). Prior to measurement, the emulsion sample was diluted with deionized water to an appropriate concentration to avoid multiple scattering effects. Fourier transform infrared (FTIR) spectra were recorded using a Nicolet iS5 spectrometer (Thermo Fisher Scientific, Waltham, MA, USA) equipped with an attenuated total reflection (ATR) accessory, with a wavenumber scanning range from 4000 cm^−1^ to 400 cm^−1^. Each spectrum was acquired by 32 scans at a spectral resolution of 4 cm^−1^. Surface morphology of the coated fabrics was observed using a Hitachi S-4800 field emission scanning electron microscope (SEM, Hitachi High-Technologies Corporation, Tokyo, Japan) at an accelerated voltage of 3.0 kV, using gold-sputtered samples to avoid charging effects. X-ray photoelectron spectroscopy (XPS) analysis was performed on an Axis-Ultra HAS system (Kratos Analytical Ltd., Manchester, UK) with 100 W Al Kα monochromatic X-ray source. A Dimension Icon atomic force microscopy (AFM, Bruker, Santa Barbara, CA, USA) working in tapping mode was used to observe the surface micromorphology and quantitatively analyze the surface roughness of the coated fabrics before and after the incorporation of SiO_2_ NPs.

Static water contact angle (WCA) was measured with a DSA100 contact angle meter (KRÜSS GmbH, Hamburg, Germany) using water drops of 6 μL. Average values were obtained by measuring at least five different positions of the samples. Water shedding angle (WSA) was tested by dropping water from a height of 1 cm to inclined fabric surface, and the minimum inclination angle of the fabric surface for water drops to completely roll off was taken as the WSA [39]. Anti-graffiti properties were tested using a marker pen to graffiti on the surface of coated fabrics, then wiping the graffiti with a wet wipe.

The washing durability of the coating was evaluated by laundering the fabric samples in a washing machine (EBM30-R198 Haier, Qingdao, China) at room temperature. Each laundering cycle consisted of 20 min of washing followed by 5 min of spinning. A commercial detergent containing nonionic and anionic surfactants and pH buffer was used, with approximately 10 g of detergent added for each washing cycle. After each cycle, the samples were rinsed with deionized water and subsequently dried in oven at 80 °C. The abrasion durability of the coating was tested according to the GB/T 3920-2008 standard [40]. An untreated fabric was used as the rubbing cloth and attached to the circular friction head of a rubbing fastness tester (model Y751B). The coated fabric sample was mounted on the testing platform, and the rubbing test was carried out for a specified number of cycles. After completion, the WCA of the fabric sample was measured.

Air permeability was evaluated according to GB/T 5453-1997 standard [41] using a fully automatic air permeability tester (YG461G, Ningbo Textile Instrument Factory, Ningbo, China). A 20 cm × 20 cm specimen was securely clamped onto the test head, and measurements were conducted at ten different locations after setting the required parameters. The average of the ten measurements was taken as the air permeability of the specimen.

## 3. Results and Discussion

### 3.1. Properties of the LSSiU Emulsions

**Figure 1:**
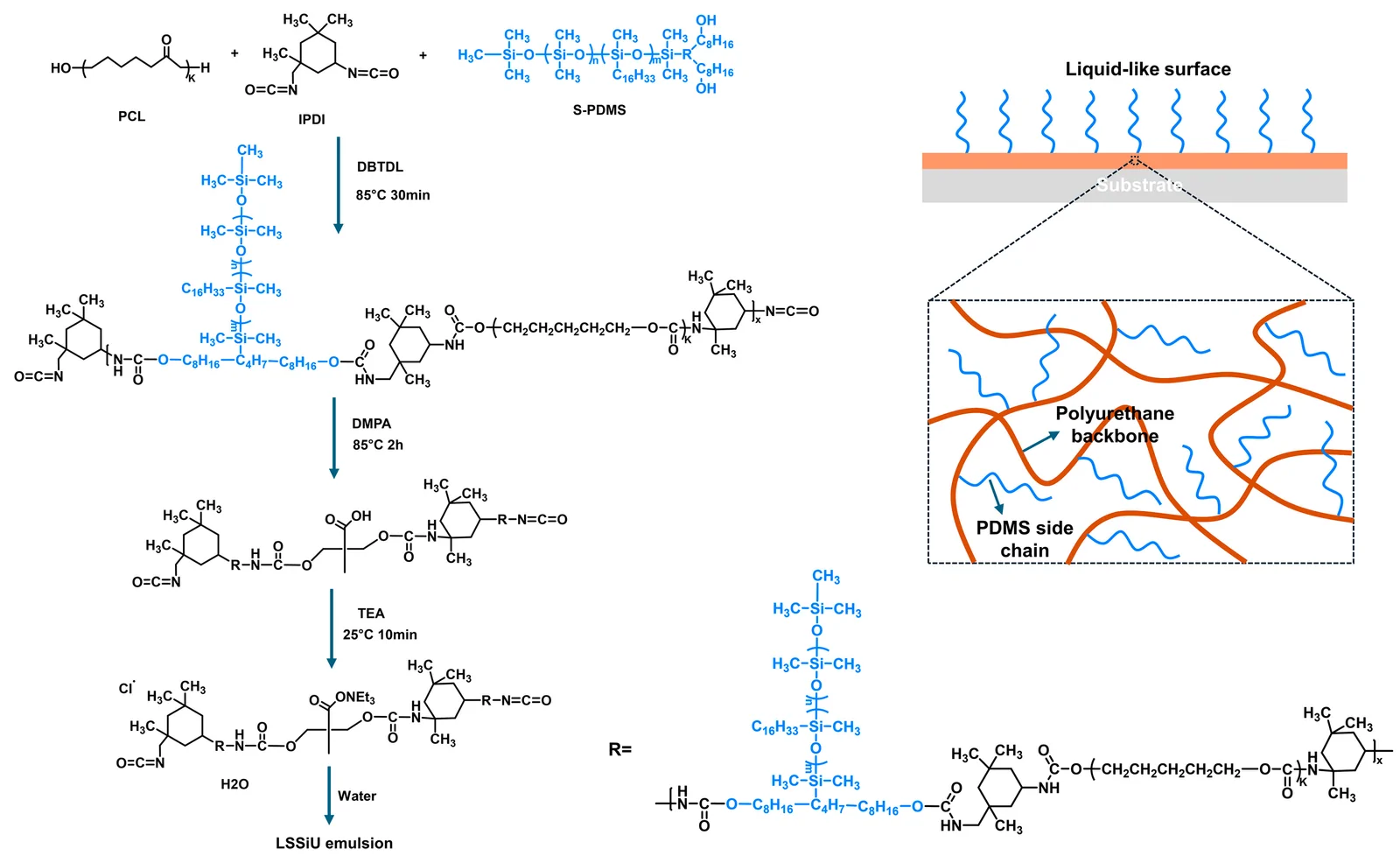
Synthesis route of the LSSiU emulsions and schematic illustration of the molecular structure consisting of polyurethane backbone and PDMS side chains.

Waterborne polyurethane emulsion stands out as an environmentally friendly alternative to solvent-based systems, with advantages including low volatile organic compound emissions, non-toxicity, and safety during handling. In addition, the molecular structure of polyurethane is highly tailorable, allowing versatile modifications through copolymerization. When applied to fabrics, polyurethane coatings offer advantages such as improved adhesion, abrasion resistance, chemical resistance, and flexibility, and with tailored formulations, they can impart fabrics with various functions (e.g., antimicrobial, flame-retardant, and antistatic effects), which makes waterborne polyurethane emulsion a highly versatile solution for advanced textile finishing [42]. In this work, we design a waterborne polyurethane emulsion for superhydrophobic and anti-graffiti fabric coating applications. Figure 1 shows the synthesis procedure of the waterborne polyurethane emulsion. In the formulation, PCL was used as the hydrophobic soft segment, as it not only provides chain flexibility for film formation but also brings intrinsic hydrophobicity that contributes to the overall water repellency of the cured coating. IPDI served as the hard segment building block, endowing the polymer network with mechanical strength and structural integrity. S-PDMS that bears two hydroxyl groups at its one end was chemically grafted into the polyurethane backbone via the reaction between its terminal hydroxyl groups and the free isocyanate moieties of IPDI. During coating formation, the flexible PDMS side chains are thermodynamically driven to migrate toward the coating-air interface because of their low surface energy. Once enriched at the surface, these flexible PDMS chains arrange themselves into a brush-like architecture, effectively imparting a liquid-like, low-adhesion surface that resists contaminant adhesion.

**Figure 2:**
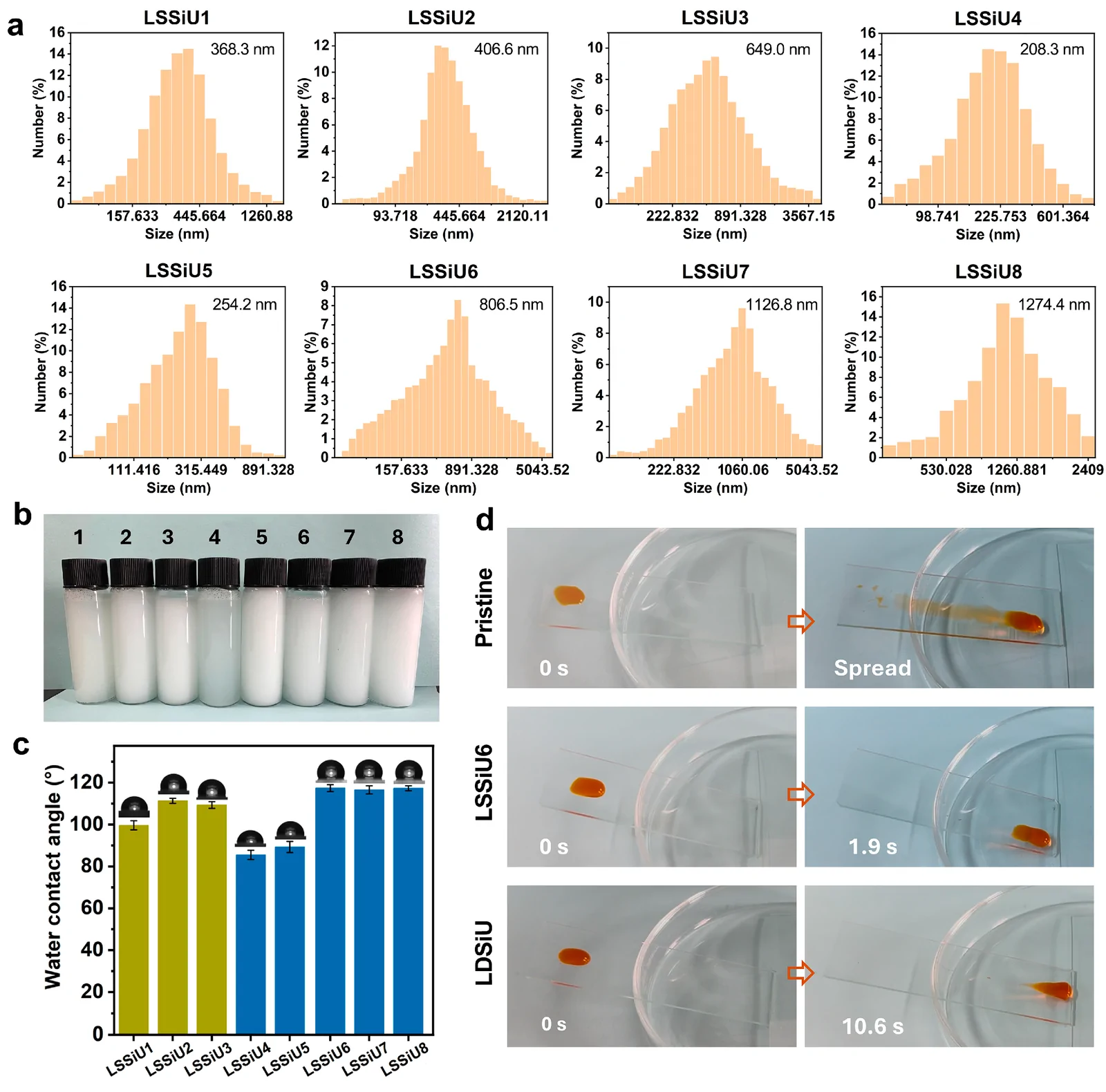
(**a**) Particle size distribution of the different LSSiU emulsions; (**b**) optical images of the different LSSiU emulsions (from left to right: LSSiU1, LSSiU2, LSSiU3, LSSiU4, LSSiU5, LSSiU6, LSSiU7, and LSSiU8); (**c**) WCAs of the glass slides coated with different LSSiU emulsions (yellow-green columns: varying DMPA content at a constant PCL/S-PDMS molar ratio; blue columns: varying the PCL/S-PDMS molar ratio at a constant DMPA content); (**d**) sliding behavior of water droplets from the top to the bottom on inclined pristine, LSSiU6-coated, and LDSiU-coated glass slides, respectively.

Figure 2a shows the particle size distribution of the obtained emulsions. For samples LSSiU1, LSSiU2, and LSSiU3, which were prepared by varying the amount of chain extender DMPA while keeping the molar ratio of PCL to S-PDMS constant, the results indicate that a higher DMPA content led to a larger particle size. This is because that the increasing DMPA content led to a rise in polymer viscosity and enhanced chain rigidity, which hindered effective shear dispersion during emulsification and offset the refining effect of increased hydrophilicity, thus resulting in larger emulsion particle sizes. When the S-PDMS content was varied, the emulsion particle size was found to increase with increasing S-PDMS content. This can be attributed to the enhanced hydrophobicity and increased viscosity imparted by PDMS, which impede the efficient breakup of polymer aggregates during emulsification. Figure 2b presents optical photographs of the LSSiU emulsions. The LSSiU4 emulsion exhibited a translucent appearance, whereas the other emulsions were milky white. For LSSiU8, a small amount of precipitate formed upon standing, suggesting a decrease in emulsion stability due to the increase in the emulsion particle size.

To study the intrinsic hydrophobicity of the LSSiU coatings, the LSSiU emulsions were spin-coated onto glass slides to form films, and the static WCAs on the coated glass surfaces were measured. As shown in Figure 2c, at a relatively low S-PDMS content, the WCA of the LSSiU4 coating was 85.5°. As the content of S-PDMS increased, the WCA of the LSSiU coating continuously increased, reaching 117.4° for the LSSiU6 coating. Further increasing the S-PDMS content led to no obvious increase in the hydrophobicity. Therefore, LSSiU6 emulsion was chosen for the following studies.

A typical feature of liquid-like surfaces is that water droplets can slide rapidly on a slightly tilted surface, owing to the high chain mobility of the grafted polymer chains [28]. To evaluate the sliding performance of water droplets on the LSSiU6 surface, the pristine and LSSiU6-coated glass slides were placed at the same inclination angle, and water droplets were deposited onto the same starting point on each slide. The time required for each droplet to slide from the top to the bottom was recorded. Figure 2d presents a comparison of the sliding speeds of water droplets on the pristine glass slide and LSSiU6-coated glass slide. When a water droplet (40 μL) was placed on the untreated glass, the surface exhibited hydrophilicity, leaving a distinct water trail as it slid through. In contrast, the droplet on the LSSiU6-coated glass slide slid rapidly without leaving a residue, and it took only 1.9 s to reach the bottom of the glass slide, demonstrating the liquid-like, lubricating effect of the PDMS side chains.

To compare the difference between single-end-anchoring and double-end-anchoring of PDMS chains on the water sliding behavior, S-PDMS in the LSSiU6 formulation was replaced with D-PDMS to prepare the LDSiU emulsion, which was then coated onto glass slide. As shown in Figure 2d, the LDSiU-coated glass slide showed hydrophobicity, but the droplet slid down at a slower pace, taking 10.6 s to reach the bottom and leaving several small water beads along the path. These results indicate that the single-end-anchored PDMS chains impart lower surface adhesion to the coating, thereby facilitating easier droplet sliding on the surface.

### 3.2. Preparation of the Superhydrophobic Fabrics

**Figure 3:**
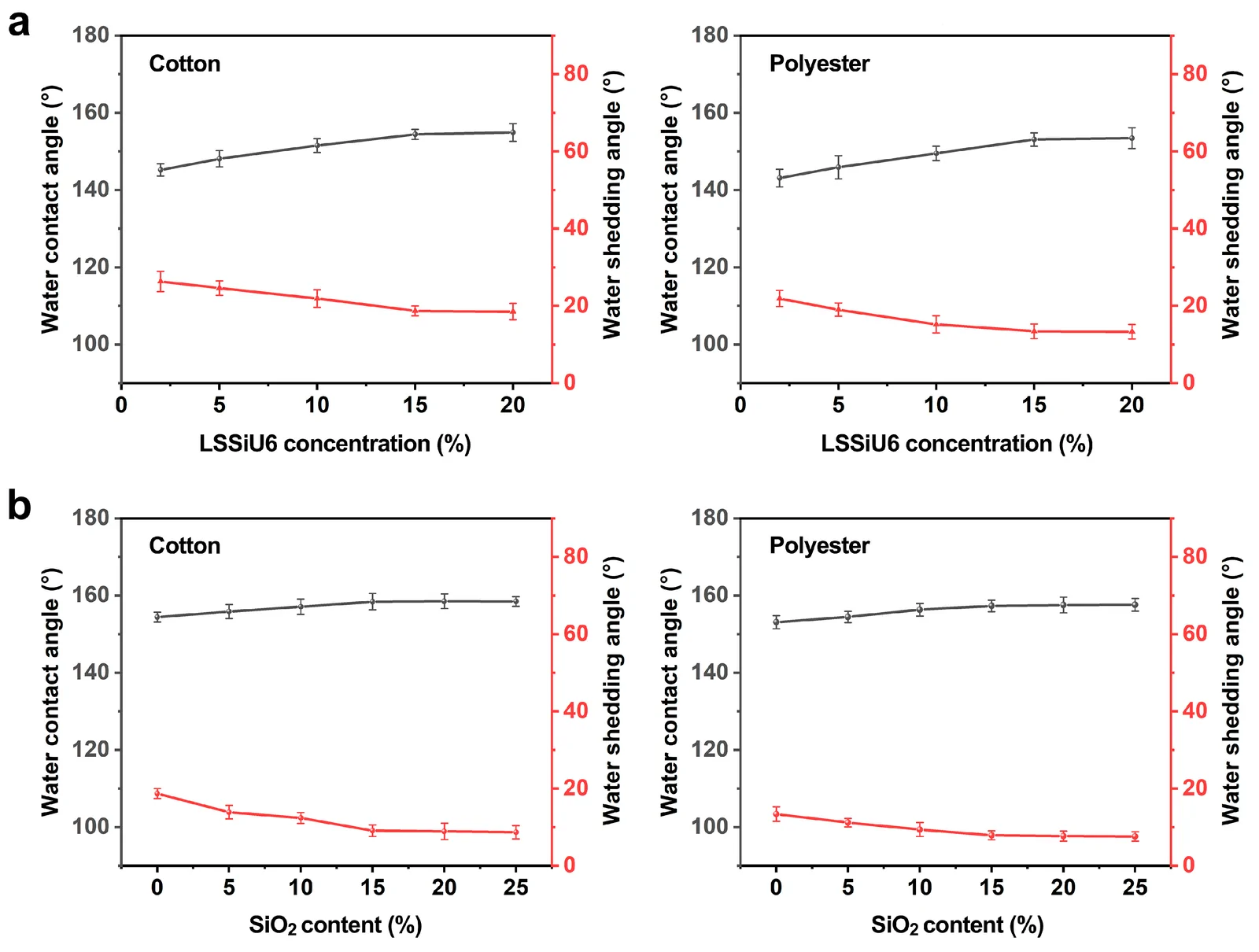
Effects of (**a**) the concentration of LSSiU6 emulsion and (**b**) the content of SiO_2_ NPs on the WCA and WSA of coated cotton and polyester fabrics.

Pristine cotton fabric is highly hydrophilic, resulting in undetectable WCA and WSA values. Although pristine polyester fabric exhibits high hydrophobicity, with a WCA of ~140°, the water droplets remained firmly adhered to the fabric surface when the sample was tilted to the vertical position, making the WSA undetectable. To prepare the superhydrophobic fabrics, the pristine fabrics were treated with LSSiU6 emulsion at different solid contents. Their WCAs and WSAs were measured and presented in Figure 3a. At a solid content of 2%, the WCAs of the LSSiU6-coated cotton and polyester fabrics were 145.2° and 143.1°, with sliding angles of 26.3° and 21.9°, respectively. As the solid content increased, the hydrophobic performance of both coated fabrics gradually improved. When the solid content reached 15%, the WCAs of the LSSiU6-coated cotton and polyester fabrics increased to 154.4° and 153.1°, while the WSAs decreased to 18.7° and 13.4°, respectively. Further increasing the solid content did not result in a significant additional improvement in hydrophobicity. Therefore, the optimal solid content of the LSSiU6 emulsion was determined to be 15%.

To further enhance the superhydrophobicity of the coated fabrics, SiO_2_ NPs were added into the LSSiU6 emulsion to increase the surface roughness of the coating. As shown in Figure 3b, with increasing SiO_2_ NPs content in the coating, the hydrophobic performance of both LSSiU6-coated cotton and polyester fabrics improved significantly. When the SiO_2_ NPs content reached 15%, the WCAs of the LSSiU6/NPs-coated cotton and polyester fabrics increased to 158.4° and 157.3°, while the WSAs decreased to 9.1° and 7.9°, respectively. The WSAs lower than 10° indicate that the as-prepared coatings exhibit slippery superhydrophobic behavior, allowing rapid droplet detachment even at minimal inclination angles, which is important for achieving the self-cleaning effect.

**Figure 4:**
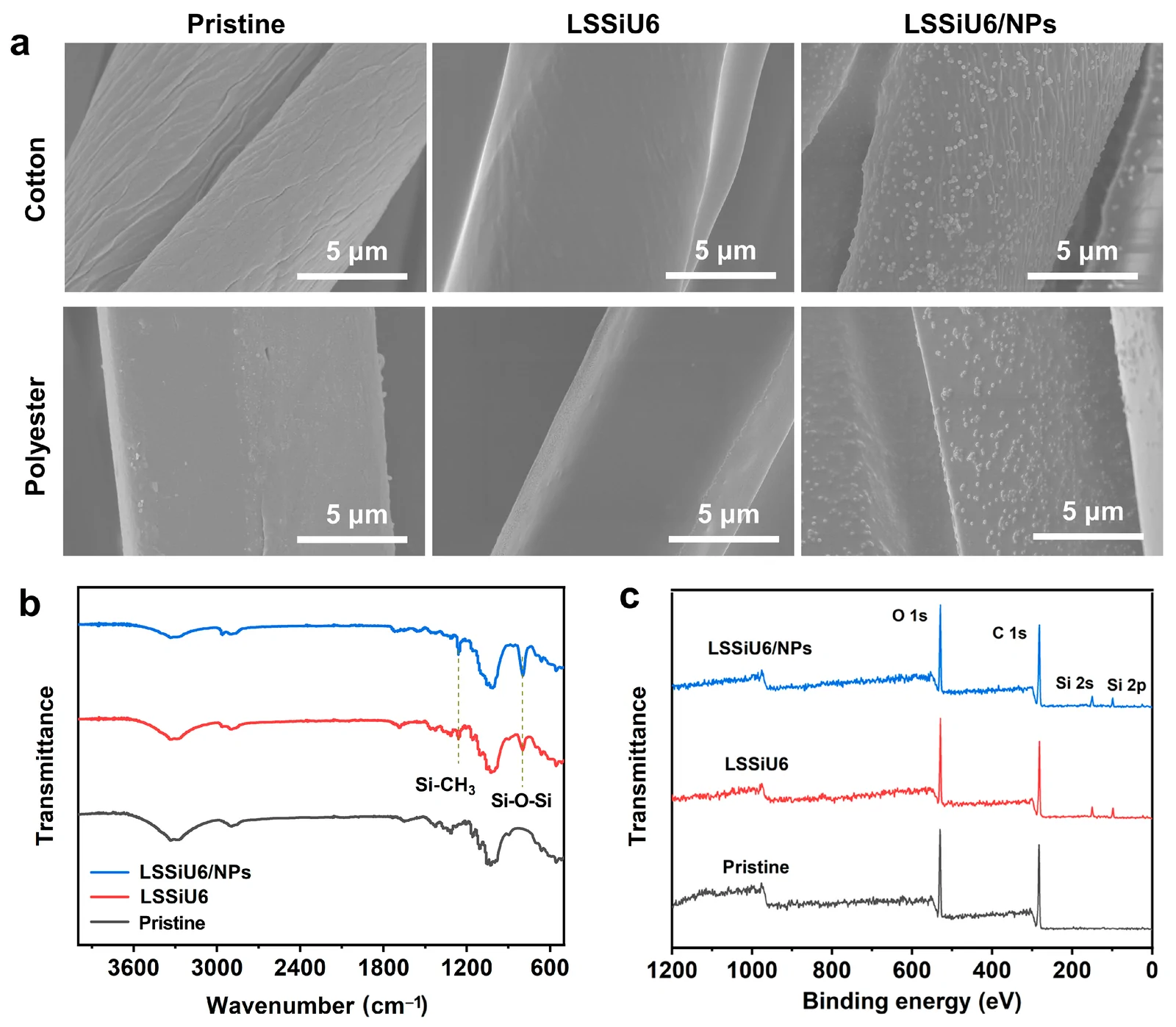
(**a**) SEM images of pristine, LSSiU6-coated, and LSSiU6/NPs-coated cotton and polyester fabrics; (**b**) FTIR and (**c**) XPS spectra of pristine, LSSiU6-coated, and LSSiU6/NPs-coated cotton fabric.

As shown in Figure 4a, both LSSiU6-coated cotton and polyester fabrics exhibited uniform and smooth films on the fiber surfaces. After the incorporation of SiO_2_ NPs into the coating, the coated fiber surfaces revealed a uniform distribution of SiO_2_ NPs, which effectively increased the surface roughness of the fibers. Fourier transform infrared (FTIR) spectroscopy was performed to investigate the changes in surface chemical composition before and after coating. As shown in Figure 4b, compared with the untreated fabrics, the most pronounced difference was the appearance of absorption band at 790–820 cm^−1^ for both the LSSiU6-coated and LSSiU6/NPs-coated fabrics. These peaks were assigned to the symmetric stretching vibration of Si–O–Si bonds originating from S-PDMS and SiO_2_ NPs. In addition, the increased intensity of this absorption band for the LSSiU6/NPs-coated fabrics could be attributed to the incorporation of SiO_2_ NPs. Furthermore, X-ray photoelectron spectroscopy (XPS) was employed to analyze the surface chemical composition. As shown in Figure 4c, the untreated cotton fabric was predominantly composed of carbon (C) and oxygen (O). After coating treatment, the S-PDMS-modified polyurethane formed a coating on the fabric surface, giving rise to two new peaks at 101.0 eV and 149.0 eV, which were assigned to Si 2p and Si 2s, respectively. Moreover, upon the incorporation of SiO_2_ NPs into the aqueous LSSiU6 emulsion, the intensities of both the Si 2p and Si 2s peaks were notably enhanced. These results well confirmed the successful deposition of the coatings onto fabric surfaces.

**Figure 5:**
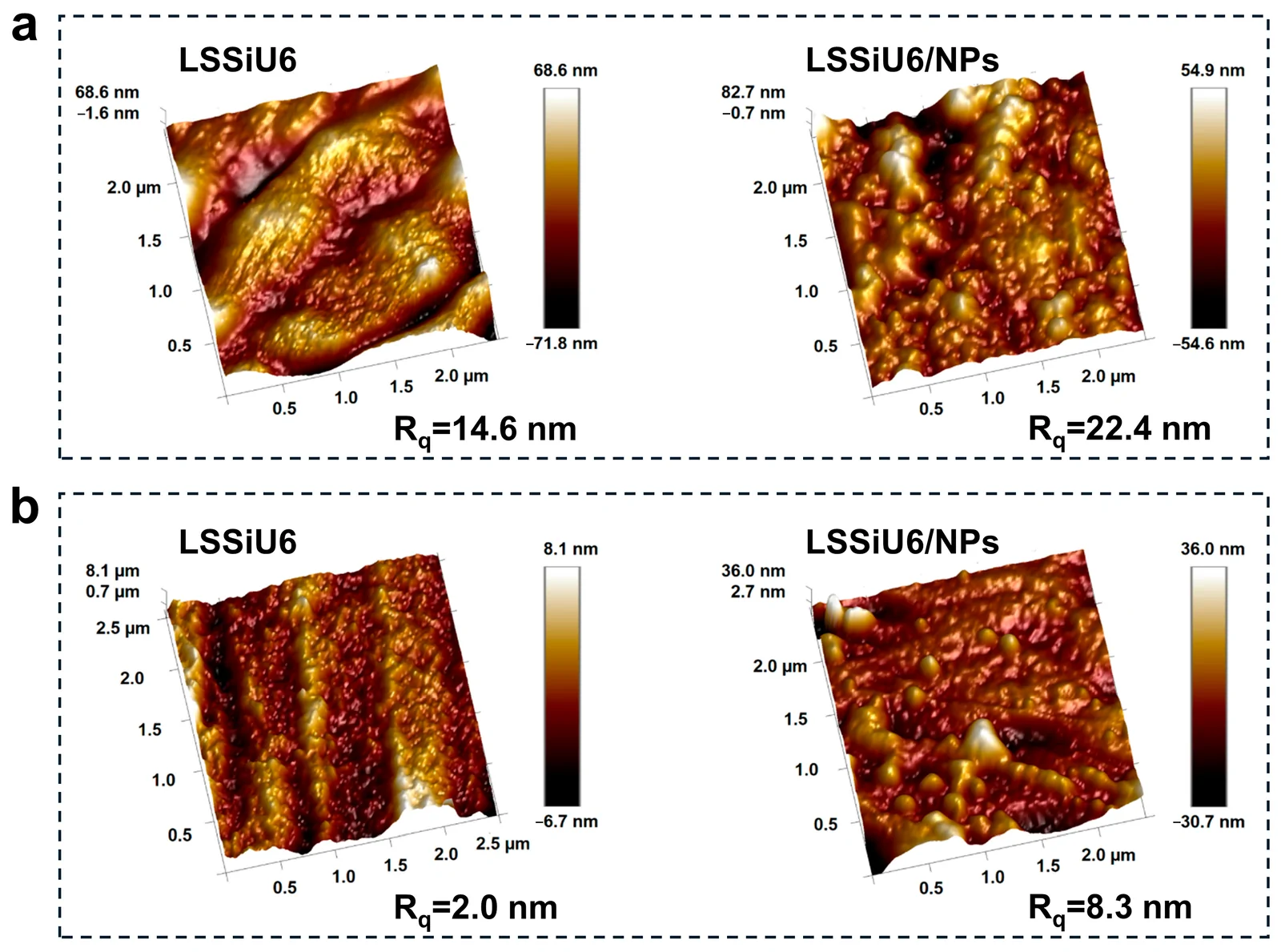
AFM images and the corresponding R_q_ values of LSSiU6-coated and LSSiU6/NPs-coated (**a**) cotton and (**b**) polyester fabrics, respectively.

In addition, AFM was utilized to examine the surface roughness variations in the coated fabrics. As shown in Figure 5, for cotton fabrics, the root-mean-square roughness (R_q_) of the LSSiU6 coating was 14.6 nm, whereas that of the LSSiU6/NPs composite coating increased to 22.7 nm. For polyester fabrics, the R_q_ values increased from 2.0 nm (LSSiU6) to 8.3 nm (LSSiU6/NPs). The introduction of SiO_2_ NPs evidently enhanced the surface roughness of the fibers for both fabric types. According to the Cassie-Baxter wetting model [43], the increased surface roughness facilitates the air entrapment at the solid–liquid interface, thus establishing a composite solid-air-liquid contact interface. In this way, the actual solid–liquid contact area and thus droplet adhesion can be reduced, thereby enabling water droplets to roll off the fabric surface more readily.

Under the optimized coating conditions, the coating add-on was 3.8 g/m^2^ and 2.4 g/m^2^ for cotton and polyester fabrics, respectively. Upon coating, the fabric flexibility slightly decreased (Figure S1), while the air permeability of cotton fabric decreased from 407 to 366 mm/s, corresponding to a 10.0% reduction, and polyester fabric decreased from 55 to 49 mm/s, corresponding to a 10.9% reduction (Figure S2). These relatively small changes suggest that the coating had a limited effect on the breathability of the fabrics. The mechanical test results (Figure S3) reveal that the coating causes a marked reduction in the breaking strength of cotton fabric due to the PDMS-induced lubrication that weakens the inter-fiber friction, while polyester remains largely unaffected due to its compact structure that confines the coating the fiber surface. Both fabrics showed reduced elongation at break because the coating layer restricts fiber and yarn movement during stretching.

### 3.3. Self-Cleaning and Anti-Graffiti Properties

**Figure 6:**
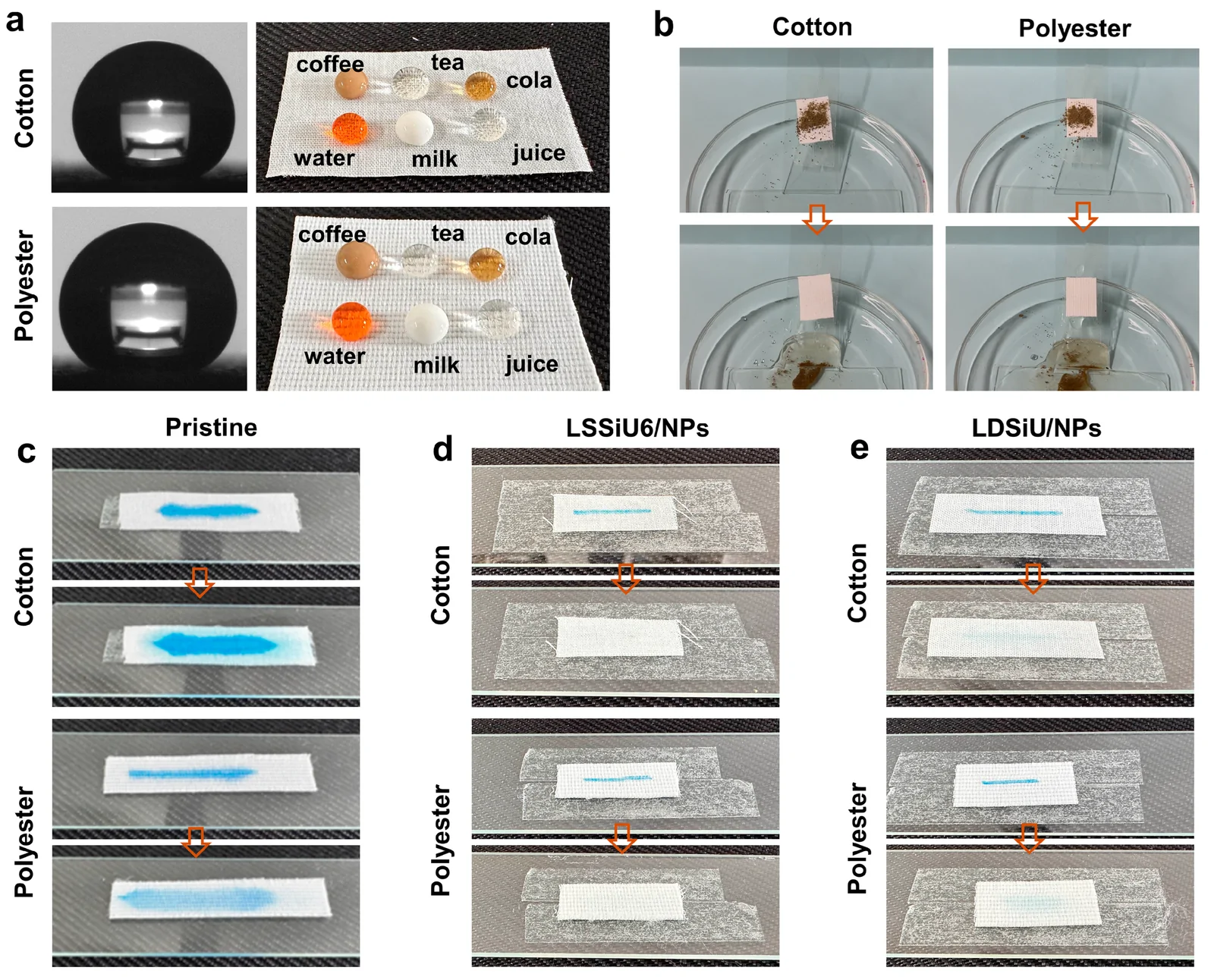
(**a**) Profiles of water droplets (**left**) and photos of various liquids (**right**) on the LSSiU6/NPs-coated cotton and polyester fabrics; (**b**) self-cleaning performance test on the LSSiU6/NPs-coated cotton and polyester fabrics; anti-graffiti performance test on the (**c**) pristine fabric, (**d**) LSSiU6/NPs-coated fabric and (**e**) LDSiU/NPs-coated fabric, respectively.

Figure 6a demonstrates the excellent water repellency of the LSSiU6/NPs-coated cotton and polyester fabrics. Water droplets placed on the coated fabrics exhibit a nearly spherical shape with a small contact area, indicating high static WCAs. When various aqueous liquids, including water, coffee, tea, cola, milk, and fruit juice, were applied onto both coated fabrics, the droplets all maintained an approximately spherical morphology with high contact angles, confirming that the LSSiU6/NPs coating endows the fabrics with excellent repellency toward a broad range of everyday liquids.

Furthermore, the self-cleaning performance of the LSSiU6/NPs-coated cotton and polyester fabrics was evaluated, and the results are shown in Figure 6b. As can be seen, upon water rinsing, the surface not only resisted wetting but also readily removed all deposited particulate contaminants (e.g., sand and dust), demonstrating that the LSSiU6/NPs coating endows the fabrics with excellent self-cleaning properties. More importantly, the LSSiU6/NPs coating imparted both cotton and polyester fabrics with excellent anti-graffiti performance. As shown in Figure 6c, on the untreated fabrics, the pigment spread readily and could not be removed away by wiping with a wet wipe. In contrast, when marker ink was applied onto the LSSiU6/NPs-treated cotton fabric (Figure 6d), it did not spread but instead remained contracted. The ink could be readily cleaned off with the same wiping procedure, leaving no visible residue. Similarly, the LSSiU6/NPs-treated polyester fabric also showed obvious ink shrinkage and effective cleanability with a wet wipe, confirming the broad substrate adaptability of the coating. Such a visual comparison provides qualitative evidence of the anti-graffiti capability of the LSSiU6/NPs coating.

To reveal the role of the single-end-anchored PDMS chains in achieving the excellent anti-graffiti performance, the control sample of LDSiU emulsion prepared by replacing S-PDMS with D-PDMS was applied onto cotton and polyester fabrics, and the anti-graffiti performance of LDSiU/NPs-coated fabrics was evaluated. As shown in Figure 6e, on the LDSiU/NPs-coated cotton and polyester fabrics, partial marker ink absorption was observed, and the ink could only be partially removed by wiping with a wet wipe. This inferior anti-graffiti performance of the LDSiU coating confirms that the single-end-anchored PDMS chains in the LSSiU6 coating contributes to the superior anti-graffiti capability.

### 3.4. Coating Durability

**Figure 7:**
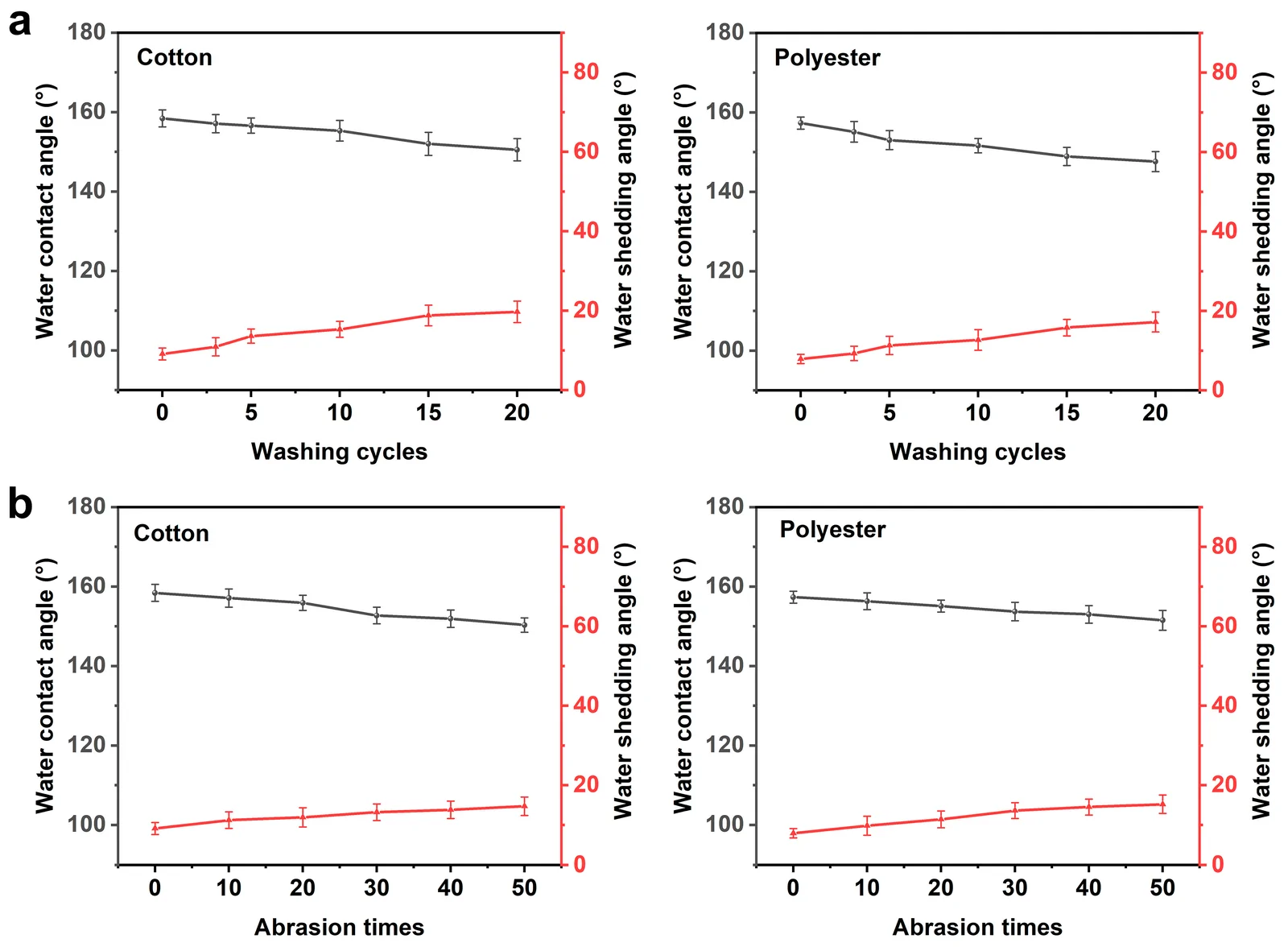
(**a**) Washing durability and (**b**) abrasion resistance of LSSiU6/NPs-coated cotton and polyester fabrics.

The mechanical durability of the coating is critical for its practical application. To evaluate the washing durability, the LSSiU6/NPs-coated fabrics were subjected to 20 laundering cycles, and the results are presented in Figure 7a. For the coated cotton fabric, the WCA decreased from 158.4° to 150.5° and the WSA increased from 9.1° to 19.7°. For the coated polyester fabric, the WCA and WSA values changed from 157.3° to 147.6° and from 7.9° to 17.2°, respectively. The increase in WSA indicates that the washing process partially weakened the water-droplet shedding ability of the coatings, which can be attributed to the surface fuzzing (Figure S4) and the resulting increase in droplet adhesion. Notably, although the WCA values remained about 150°, the WSA values exceeded 10° after washing, indicating that the fabrics no longer fully satisfy the commonly accepted criterion for superhydrophobicity. Nevertheless, the coated fabrics still retained high water repellency after washing, demonstrating that the LSSiU6/NPs coating possesses reasonable washing durability and can maintain its hydrophobic surface characteristics under routine washing conditions.

In addition, the abrasion resistance of the LSSiU6/NPs-coated fabrics was evaluated over 50 rubbing cycles (Figure 7b). For cotton, the WCA decreased from 158.4° to 151.9°, while the WSA increased from 9.1° to 15.7°. For polyester, the WCA values changed from 157.3° to 151.5°, with an increase in WSA from 7.9° to 13.5°. Besides the surface fuzzing similar to that observed in the aforementioned washing test, abrasion-induced coating damage (Figure S5) also contributed to the increase in WSA by enhancing water-droplet adhesion. Nevertheless, both fabrics retained a WCA above 150°, confirming satisfactory abrasion resistance for practical use.

To assess the chemical stability of the coating under different environmental conditions, the LSSiU6/NPs-coated polyester fabrics were immersed in pH 3 HCl solution, pH 11 NaOH solution, and 3.5 wt% NaCl solution to represent acidic, alkaline, and saline environments, respectively. After 7 h of immersion, the WCAs remained around 150° under all three conditions (Figure S6), suggesting good resistance to these chemical environments. In contrast, under relatively intense UV irradiation (320–500 nm, 30 W/m^2^), the WCA decreased below 150° within 20 s (Figure S7), indicating that UV exposure may represent a more critical factor affecting the long-term stability of the coating.

Finally, the performance and durability of various PDMS-based superhydrophobic fabrics were compared (Table 2), in terms of WCA, WSA, durability, and anti-graffiti property. As can be seen, the coating composition, fabrication method, and testing conditions vary substantially among these studies, and the comparison is intended to provide a broader context rather than a strict one-to-one comparison.

## 4. Conclusions

In summary, we have developed a waterborne, fluorine-free superhydrophobic coating for fabric substrates that combines liquid-like surface behavior with excellent anti-graffiti performance. The key to this design lies in the covalent incorporation of dihydroxy single-end-terminated PDMS into the polyurethane backbone, which not only lowers the surface free energy of the coating but also enables the spontaneous enrichment and orientation of PDMS side chains at the coating-air interface during film formation. The coating was applied to both cotton and polyester fabrics via a simple yet effective dip-coating process, yielding slippery superhydrophobicity with static WCAs of 158.4° and 157.3°, and WSAs as low as 9.1° and 7.9°, respectively. Besides the excellent water repellency, the coated fabrics exhibited anti-graffiti performance, as well as self-cleaning ability that enables easy removal of surface contaminants by water droplets. Moreover, the coating demonstrated satisfactory mechanical durability, retaining WCA about 150° after 20 laundering cycles or 50 abrasion cycles. This waterborne, fluorine-free strategy offers a practical and eco-friendly route to extend liquid-like repellent surfaces from smooth substrates to textile substrates for real-world anti-graffiti and self-cleaning applications.

## Figures and Tables

**Table 1:** Recipes used for preparing the LSSiU emulsions.

Sample	Mass (g)				
	PCL	S-PDMS	IPDI	DMPA	TEA
LSSiU1	1.50	12.75	2.40	0.40	0.36
LSSiU2	1.50	12.75	3.02	0.80	0.73
LSSiU3	1.50	12.75	4.00	1.21	1.09
LSSiU4	2.25	6.38	3.02	0.80	0.73
LSSiU5	2.00	8.50	3.02	0.80	0.73
LSSiU6	1.00	17.00	3.02	0.80	0.73
LSSiU7	0.75	19.13	3.02	0.80	0.73
LSSiU8	0.60	20.40	3.02	0.80	0.73

**Table 2:** Comparison of various PDMS-based superhydrophobic fabrics.

Materials	WCA	WSA	Washing Durability	Abrasion Resistance	Chemical Stability	Anti-Graffiti	Ref.
PDMS (Sylgard 184)/SiO_2_	155°	-	-	30 cycles (Sandpaper, 1000 mesh)	pH 1, 2, 13, 14 water and 0.9% NaCl for 1 h	-	[8]
PDMS (Sylgard 184)/ZnO	156°	4°	-	20 cycles (Sandpaper, 800 mesh)	-	-	[9]
V-PDMS/mMoS_2_	157°	6°	-	100 cycles (Sandpaper, 800 mesh)	hexane, toluene, 7 wt% NaCl, pH 1 HCl water, pH 13 NaOH water for 24 h	-	[10]
Plasma-treated PDMS	162.7°	7.5°	300 cycles (GB/T 3921-2008 [44])	600 cycles (GB/T 3920-2008)	-	-	[13]
Plasma-treated PDMS/PTFE particle	152°	-	-	-	-	-	[14]
Polyurethane with PDMS side chains/SiO_2_	157–158°	<10°	20 cycles (Conventional laundering)	50 cycles (GB/T 3920-2008)	pH 3 HCl solution, pH 11 NaOH solution, and 3.5 wt% NaCl solution for 7 h	Yes	This work

“-”: Not reported. V-PDMS: vinyl-terminated polydimethylsiloxane.

## Data Availability

Data are contained within the article and Supplementary Material. Further inquiries can be directed to the corresponding author.

## Supplementary Materials

The following supporting information can be downloaded at: https://www.mdpi.com/article/10.3390/polym18172063/s1. Figure S1. Comparison of the flexibility of fabrics before and after coating. Figure S2. Changes in the air permeability of the cotton and polyester fabrics before and after coating. Figure S3. Stress-strain curves of cotton and polyester fabrics before and after coating. Figure S4. SEM image of the LSSiU6/NPs-coated cotton fabric after washing test. Figure S5. SEM image of the LSSiU6/NPs-coated polyester fabric after abrasion test. Figure S6. Water contact angles of the LSSiU6/NPs-coated polyester fabric after immersion in pH 3 HCl water, 3.5 wt% NaCl, and pH 11 NaOH water for 7 h. Figure S7. Change in the water contact angle of the LSSiU/NPs-coated polyester fabric under the broad-spectrum UV irradiation (320–500 nm) with an intensity of ~30 W/m^2^.
