# Supplementary material for: Fluorine-Free and Waterborne: A Sustainable Polyurethane Coating Strategy for Superhydrophobic and Anti-Graffiti Fabrics
Source: Polymers (Basel). 2026 Aug 25;18(17):2063. doi: 10.3390/polym18172063 (PMC13567686; doi:10.3390/polym18172063)
Supplement: Supplementary file 1 [file polymers-18-02063-s001.zip › polymers-4491763-supplementary.pdf]

Fluorine-Free and Waterborne: A Sustainable Polyurethane Coating Strategy for Superhydrophobic and Anti-Graffiti Fabrics

Jing Yang [1], Shengzhe Hou [1], Ziyi Li [1] and Yan Zhao [1,2,*]

[1] College of Textile and Clothing Engineering, National Engineering Laboratory for Modern Silk, Soochow University, Suzhou 215123, China

[2] Jiangsu Advanced Textile Engineering Technology Center, Nantong 226007, China

* Correspondence: yanzhao@suda.edu.cn

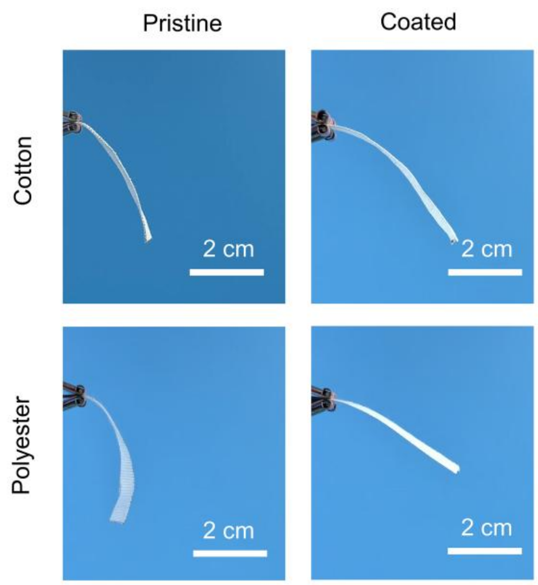

Figure S1. Comparison of the flexibility of fabrics before and after coating.

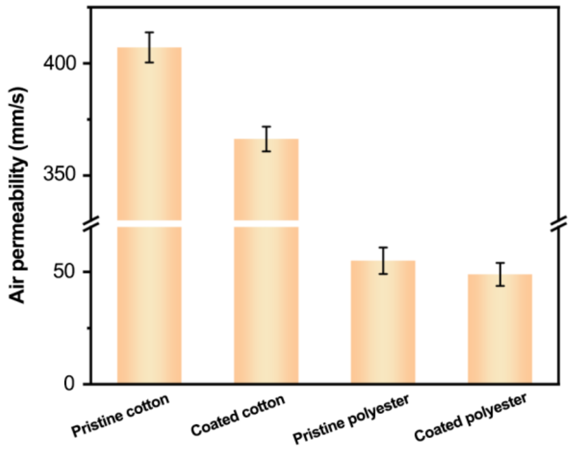

Figure S2. Changes in the air permeability of the cotton and polyester fabrics before and after coating.

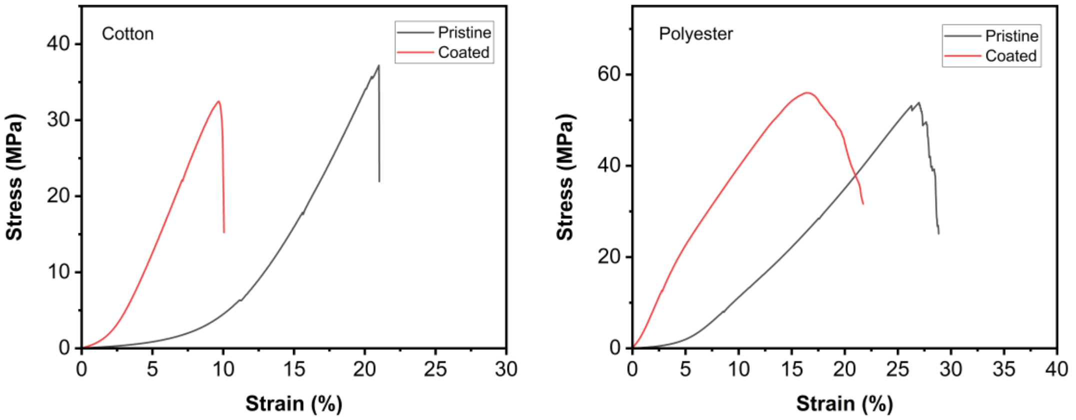

Figure S3. Stress-strain curves of cotton and polyester fabrics before and after coating.

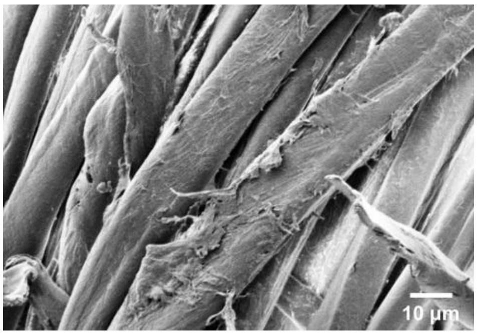

Figure S4. SEM image of the LSSiU6/NPs-coated cotton fabric after washing test.

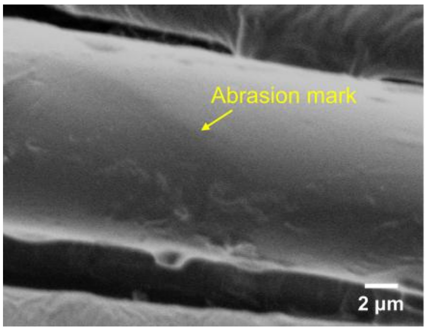

Figure S5. SEM image of the LSSiU6/NPs-coated polyester fabric after abrasion test.

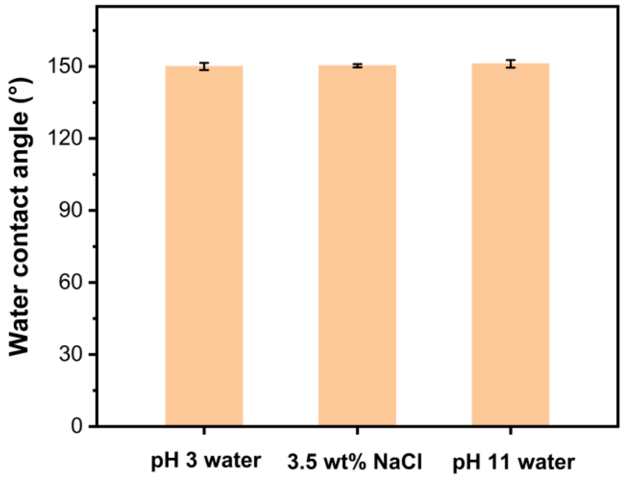

Figure S6. Water contact angles of the LSSiU6/NPs-coated polyester fabric after immersion in pH 3 HCl water, 3.5 wt% NaCl, and pH 11 NaOH water for 7 h.

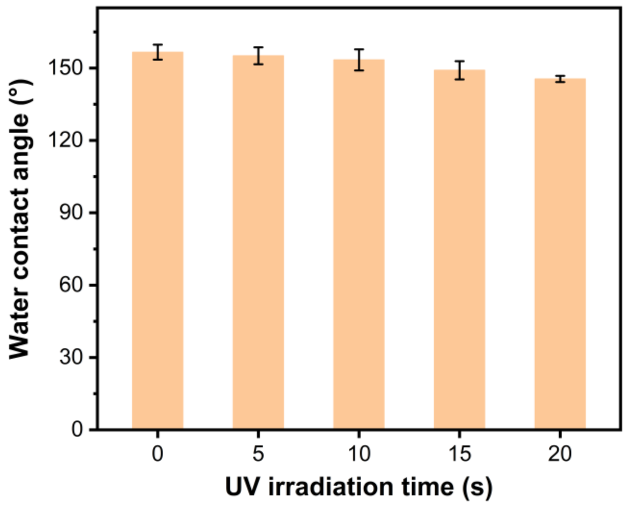

Figure S7. Change in the water contact angle of the LSSiU/NPs-coated polyester fabric under the broad-spectrum UV irradiation (320-500 nm) with an intensity of ~30 W/m^2.
